# Divergent methylation of CRISPR repeats and *cas* genes in a subtype I-D CRISPR-Cas-system

**DOI:** 10.1186/s12866-019-1526-3

**Published:** 2019-07-01

**Authors:** Ingeborg Scholz, Steffen C. Lott, Juliane Behler, Katrin Gärtner, Martin Hagemann, Wolfgang R. Hess

**Affiliations:** 1 Faculty of Biology, Genetics an Experimental Bioinformatics, University of Freiburg, Schänzlestr. 1, D-79104 Freiburg, Germany; 2 University of Rostock, Institute of Biosciences, Plant Physiology, A.-Einstein-Str. 3, D-18059 Rostock, Germany; 3 University of Freiburg, Freiburg Institute for Advanced Studies, Albertstr. 19, D-79104 Freiburg, Germany

**Keywords:** CRISPR-Cas, Cyanobacteria, DNA methyltransferase, Highly iterated palindrome 1, HIP1, DNA methylation

## Abstract

**Background:**

The presence and activity of CRISPR-Cas defense systems is a hallmark of many prokaryotic microorganisms. Here, the distribution of sequences related to the highly iterated palindrome 1 (HIP1) element and the DNA methylation of CGATCG motifs embedded within HIP1 as a vital part of the CRISPR1 repeat sequence was analyzed in the cyanobacterium *Synechocystis* sp. PCC 6803. Previously suggested functions of HIP1 include organization of chromosomal structure, DNA recombination or gene regulation, all of which could be relevant in CRISPR-Cas functionality.

**Results:**

The CRISPR1 repeat-spacer array contains more than 50 CGATCG elements that are double-methylated (^5m^CG^6m^ATCG) by the enzymes M.Ssp6803I and M.Ssp6803III. Hence, more than 200 possible methylation events cluster over a stretch of 3600 bp of double-stranded DNA. Bisulfite sequencing showed that these motifs were highly methylated at the ^m5^CGATCG positions whereas specific motifs within the CRISPR1 *cas* genes were hypomethylated suggesting a lowered accessibility for the DNA methylase to these regions. Assays for conjugation and CRISPR1-mediated DNA interference revealed a 50% drop in conjugation efficiency in the mutant lacking the ^5m^C methylation of CGATCG motifs, while the highly efficient DNA interference activity was not affected by the lack of ^m5^CGATCG DNA-methylation, nor was the capability to differentiate between self and non-self targets based on the protospacer adjacent motifs (PAMs) GTA and GTC versus the non-PAM AGC. A third DNA methylation mediated by M.Ssp6803II modifies the first cytosine in the motif GGCC yielding GG^m4^CC. We found a remarkable absence of GGCC motifs and hence the corresponding methylation over an 11 kb stretch encompassing all the *cas* genes involved in interference and crRNA maturation but not adaptation of the CRISPR1 system.

**Conclusions:**

The lack of GGCC tetranucleotides along the CRISPR1 interference and maturation genes supports the reported hybrid character of subtype I-D CRISPR-Cas systems. We report tight and very high ^5m^C methylation of the CRISPR1 repeat sequences. Nevertheless, cells lacking the ^5m^C methylation activity were unaffected in their CRISPR1-mediated interference response but the efficiency of conjugation was reduced by 50%. These results point to an unknown role of ^m5^CGATCG DNA-methylation marks in conjugation and DNA transformation.

## Background

The highly iterated palindrome 1 (HIP1) element 5′-GCGATCGC-3′ is an octameric palindromic repeat that is overrepresented in several cyanobacteria [1, 2]. In the chromosome of the cyanobacterial model *Synechocystis* sp. PCC 6803 (from here: *Synechocystis* 6803) HIP1 instances occur at the frequency of one copy in every 1131 bp [1, 3]. Statistical analyses supported the hypothesis that HIP1 motifs are maintained by selection, suggesting that HIP1 motifs likely perform biological functions [4]. A relation between the presence of HIP1 motifs and DNA recombination and/or repair processes has been suggested [5]. In addition or alternatively, a potential HIP1 function associated with chromosomal structure or maintenance was suggested based on its distribution along the chromosome [4].

At its core, the HIP1 element contains the recognition sequence of Dam DNA methyltransferases. These N6-adenine-specific enzymes modify the adenosine residue within the target sequence GATC and are often essential for viability [6]. Methylation at the position G^m6^ATC in *Synechocystis* 6803 is carried out by the DNA methyltransferase M.Ssp6803III encoded by gene *slr1803*, which was found to be essential for viability of this cyanobacterium [7]. Moreover, the first cytosine within the HIP1 sequence is ^m5^C-methylated in *Synechocystis* 6803 by the DNA methyltransferase M.Ssp6803I encoded by *slr0214* [7, 8]. Hence, in this cyanobacterium, the hexanucleotide 5′-CGATCG-3′ within the HIP1 element can be methylated at four individual positions on the two DNA strands. Similar methylation patterns of HIP1 sequences have been reported for *Anabaena* sp. PCC 7120 [9]. In addition, the DNA methyltransferase M.Ssp6803II, encoded by *sll0729*, was recognized to methylate the first cytosine in the frequently occurring motif GGCC at the ^m4^C position yielding GG^m4^CC [7]. GGCC is the most frequent methylation motif in *Synechocystis* 6803, on average providing one methylation site every 185 bp on the chromosome.

Clustered regularly interspaced short palindromic repeats (CRISPRs)-Cas systems are adaptive immune systems in bacteria and archaea that use CRISPR RNAs (crRNAs) as guides and CRISPR-associated proteins (Cas) for antiviral defense [10–13]. There are three different CRISPR-Cas systems in *Synechocystis* 6803 [14]. Based on the associated *cas* gene complement, these systems were classified as one subtype I-D (CRISPR1), one subtype III-D (CRISPR2) and one subtype III-Bv (CRISPR3) CRISPR-Cas system [14, 15].

The crRNAs originate from the CRISPR repeat-spacer arrays initially in the form of long precursor transcripts. After transcription, the CRISPR repeats are recognized by processing maturases. These frequently belong to the Cas6 class of endoribonucleases [16] whereas in subtype I-C systems the endoribonuclease is Cas5d [17, 18]. In case of *Synechocystis* 6803, crRNA maturation proceeds by the Cas6–1 enzyme for the CRISPR1 system and by Cas6-2a for the CRISPR2 system [14, 19, 20], while for the CRISPR3 system RNase E was recognized as the major maturation endoribonuclease [15]. During interference, crRNAs guide the proteinaceous CRISPR effector complex to their targets, also known as protospacers, resulting in efficient immunity against potentially harmful invading nucleic acids [21–23]. CRISPR1 interference activity was shown to strictly depend on the presence of a DNA sequence element called protospacer adjacent motif (PAM). PAM sequences are functionally critical for CRISPR-based immune systems and are located adjacent to each protospacer consisting of a short signature sequence of 2–5 nt, depending on the CRISPR type and organism. The PAM sequences GTN were found to efficiently mediate CRISPR1 interference in *Synechocystis* 6803, while there also exist PAMs, e.g. the sequence motif AGC, that do not license interference [24].

While unexpected connections between bacterial natural competence, ubiquitin signaling and DNA modification was reported for type VI-B CRISPR-Cas systems [25], a possible relationship between DNA methylation and CRISPR-Cas-mediated interference responses has not been studied thus far. We noticed the CGATCG sequence to be overrepresented within the CRISPR1 system of *Synechocystis* 6803, with possibly more than 200 methylation events over a stretch of only 3600 nt. The recent availability of bisulfite sequencing data for this organism [7] enabled the detailed analysis of pSYSA cytosine methylation. Therefore, here we investigated the DNA methylation of the CRISPR1 system within the context of the pSYSA plasmid and whether there was a correlation between DNA methylation and DNA interference in a conjugation-based assay using a DNA methyltransferase mutant.

## Methods

### Strains and culture conditions

*Synechocystis* sp. 6803 was maintained on BG11 mineral medium [26] at 30 °C under constant illumination. The *slr0214* deletion mutant (Δ*slr0214*-A1) and the *slr0214* insertion mutant (Δ*slr0214*-B1) were described previously [7, 8]. Liquid cultures of *Synechocystis* 6803 wild type and *slr0214* mutants were grown photoautotrophically in volumes of 50 mL in Erlenmeyer flasks in BG11 medium, with shaking at 50 μmol photons s^− 1^ m^− 2^ at 30 °C. *E. coli* cultures were grown in LB medium at 37 °C. Growth was followed by measurements of the optical density at 750 nm (OD_750_) for *Synechocystis* 6803 and at 600 nm (OD_600_) for *E. coli*.

### *Synechocystis* 6803 conjugation and interference assay

Conjugation between *E. coli* and *Synechocystis* 6803 by triparental mating was essentially carried out as described previously [14]. In short, flasks containing LB medium without antibiotics were inoculated with overnight cultures of *E. coli* J53/RP4 (helper strain) and DH5α with the plasmid of interest (donor strain) to obtain an OD_600_ of 0.1 and incubated at 37 °C with shaking at 180 rpm for 2.5 h. Plasmid-bearing and helper strains equivalent to an OD_600_ of 7.0 were harvested by centrifugation, resuspended in 1 mL LB, combined (1 mL plasmid-bearing and 1 mL helper strain), centrifuged again, resuspended in 100 μL LB and incubated at 30 °C without shaking for 1 h. In parallel, cultures of the *Synechocysti*s 6803 wild type and *slr0214* deletion strains [7] equivalent to an OD_750_ of 0.75 were harvested by centrifugation, resuspended in 800 μL BG11 medium and combined with the *E. coli* cells. The resulting mix was centrifuged, resuspended in 30 μL BG11 and placed on a sterile filter located on a BG11 agar plate supplemented with 5% LB medium at 30 °C overnight (slightly covered with tissue). The next morning the filter was rinsed with 400 μL BG11 medium, 20 μL and 40 μL of the cell suspension were plated, respectively, on BG11 plates containing 5 μg mL^− 1^ gentamicin.

Interference assays essentially were performed as described [15, 24] using tri-parental mating with the self-replicating conjugative vector pVZ322 [27] containing the gentamicin resistance cassette for selection plus a protospacer sequence to spacer 1 of the CRISPR1 system with either of the PAMs GTA, GTC or the non-PAM AGC as a control. These plasmids were constructed during previous work [24].

The *Synechocysti*s 6803 wild type, the *slr0214* deletion strain (Δ*slr0214*-A1) and the *slr0214* insertion strain (Δ*slr0214*-B1) [7] were used as recipients. Transconjugant colonies were counted after incubation at 30 °C for 2 weeks. Experiments were performed in biological triplicates for the plasmid targets (pT) and in parallel with the control plasmids (pNT) either without protospacer sequence or containing the AGC non-PAM fused to a protospacer.

### Methylation analysis by bisulfite sequencing

The bisulfite sequencing data were obtained in the frame of a previous study [7]. In short, ~ 200 ng of DNA were bisulfite treated with the Zymo Gold kit (Zymo Research, Cat. No. D5005) and libraries constructed using the Ovation Ultra-Low Methyl-Seq library kit (NuGEN Cat. No. 0535–32) according to the manufacturer’s instructions, followed by sequencing on the Illumina HiSeq2500 system yielding 2,559,017 raw reads. The sequences were-quality filtered and adapter-trimmed using Trimmomatic v0.36 [28] and FastQC v0.67 (http://www.bioinformatics.babraham.ac.uk/projects/fastqc/) leaving 2,552,913 reads for further analysis. For mapping to the *Synechocystis* 6803 pSYSA plasmid and quantitative evaluation, Bismark v0.17 was used with default options [29] in conjunction with Bowtie 2 [30]. The raw sequencing data can be accessed at https://www.ncbi.nlm.nih.gov/biosample/8378604 (BioProject ID: PRJNA430784, BioSample: SAMN08378604, Run: SRX3574087).

### DNA manipulation and hybridization

For total DNA extraction from the *Synechocystis* 6803 wild type, Δ*slr0214*-A1 and Δ*slr0214*-B1 strains, cultures of 50 mL were harvested by centrifugation for 5 min. The pellet was resuspended in 1 mL SET buffer (50 mM Tris, pH 7.5; 1 mM EDTA, pH 8; 25% (w/v) sucrose) and shock frozen in liquid nitrogen. For lysis, cells were incubated overnight at 50 °C in 100 mM EDTA (pH 8), 2% SDS (w/v) and 100 μg/mL proteinase K. After lysis, DNA was extracted twice by adding 1 vol phenol/chloroform/isoamylalcohol (25:24:1) and once by adding 1 vol chloroform/isoamylalcohol (24:1). After each addition, the solution was mixed and phase separation was achieved by centrifugation (6 min, 6000 rpm) in a swing-out rotor. The upper aqueous phase was removed and DNA was precipitated by addition of 1 vol isopropanol and incubation at − 20 °C overnight. DNA was collected by centrifugation (13,000 rpm) for 30 min at 4 °C. The pellet was washed with 70% ethanol, air-dried and resuspended in 50 μL sterile Milli-Q water. DNA concentration was measured in a NanoDrop spectrophotometer (ND-1000, peQLab). The quality and quantity of nucleic acid extraction was verified optically in ethidium bromide stained 0.8% agarose gels.

For restriction analysis, 10 μg each of total DNA from *Synechocystis* 6803, Δ*slr0214*-A1 and Δ*slr0214*-B1 were digested with *Pvu*I, *Dpn*I or *Sau*3AI. Restriction endonucleases were used in a 10-fold excess and incubated overnight at 37 °C to ensure complete digestion. Heat inactivation was performed at 80 °C (*Pvu*I and *Dpn*I) and 65 °C (*Sau*3AI) for 20 min. Digested total DNA was separated overnight on an ethidium bromide stained 1.2% agarose gel under a field strength of 1.8 V cm^− 1^ at 4 °C. The gel was incubated in denaturation solution (1.5 M NaCl, 0.5 M NaOH) at 70 rpm at room temperature for 30 min and subsequently in neutralization solution (1.5 M NaCl, 0.5 M Tris pH 7.5) for the same time. DNA was transferred to Hybond-N+ membranes (Amersham Cat. No. RPN303B) with 10x saline sodium citrate (3 M NaCl, 300 mM sodium citrate pH 7) transfer buffer by capillary blotting overnight. After blotting the DNA was crosslinked to the membrane with 125 mJ using a UV-Stratalinker (Stratagene).

The following synthetic primers were used to generate the DNA probe for Southern hybridization by PCR: SS_slr7010_fw, 5`-CCAAGAACGTCAGCAAACCCAAAC-3′ and SS_slr7010_rev, 5′-CCATCCCAAATCCCTGACTGTAAAG-3′. PCR amplification was performed with Q5 high fidelity DNA polymerase (NEB Cat. No. M0491S) (0.02 U μL^− 1^) at 50 μL scale, containing 10 ng of template DNA, 200 μM dNTPs, 0.5 μM of each primer and 10 μL of 5x Q5 reaction buffer. The initial denaturation temperature was 98 °C for 30 s. Thirty-two cycles were performed at 98 °C for 10 s, 60 °C for 30 s and 72 °C for 18 s, followed by a 2 min final extension at 72 °C. The PCR product was loaded on a Midori green (Nippon Genetics Cat. No. MG04) stained 1.8% agarose gel and the correct band was gel-eluted using the gel extraction kit from Macherey-Nagel (Cat. No. 740609.10).

For Southern hybridization, 25 ng of DNA probe was labeled with α^32^P-dCTP using the Random Primers DNA Labeling System from Thermo Fisher Scientific (Cat. No. 18187013). Hybridization was performed overnight at 58 °C in Southern hybridization buffer (250 mM NaPi-buffer pH = 7.2, 7% SDS, 250 mM NaCl) followed by 10 min wash steps each in wash buffers 1 (2x SSC, 1% SDS), 2 (1x SSC, 0.1% SDS) and 3 (0.1x SSC, 0.1% SDS) at 53 °C. The signals were detected with a storage phosphor screen (Kodak) and a GE Healthcare Typhoon FLA 9500 imaging system.

## Results

### Distribution and methylation of GGCC motifs on the pSYSA plasmid

**Fig. 1:**
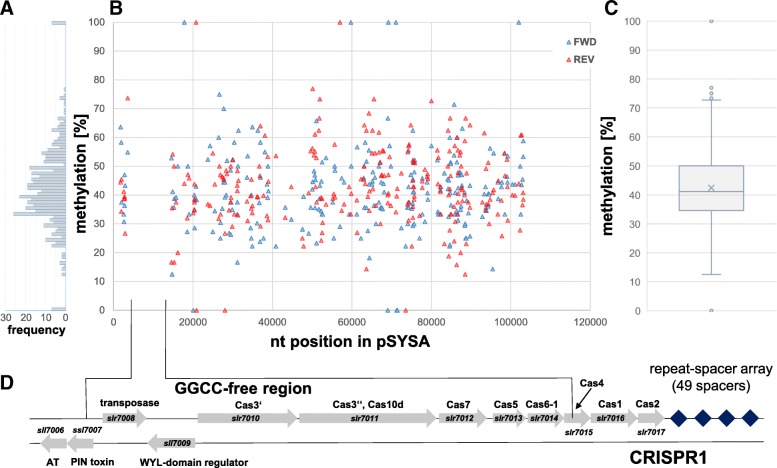
Distribution and bisulfite analysis of the methylation status of GGCC motifs on the plasmid pSYSA. **a** Frequency of detecting methylated GG^m4^CC motifs at the give percentage range in panel (B). **b** GGCC methylation sites are plotted as blue triangles (forward strand) and red triangles (reverse strand) along a linear plot of the pSYSA sequence. In the *Synechocystis* 6803 reference strain pSYSA is 103,307 nt long [51]. The bisulfite-measured percentage of methylation is plotted along the Y axis. **c** Average measured GG^4m^CC methylation. For the underlying data, the cross in the box represents the mean (42.5%) and the line close to the cross shows the median (41.2%). The box itself contains 50% of the data (25% quartile – 75% quartile), while the whiskers are 1.5 of the interquartile range (IQR). Data points above the upper whisker as well as data points below the bottom whisker are outliers. **d** The GGCC-free region from positions 3537 to 14,544 is enlarged. The gene identifiers are given in italics and the encoded proteins above and below the respective genes. The CRISPR1 region begins with gene *sll7009* und finishes with the repeat-spacer region. This figure is a visualization of the results obtained in this work using standard software (Microsoft Excel 2016 and Microsoft Power Point 2016)

We used the bisulfite sequencing data that were obtained in the frame of the global characterization of DNA methyltransferases in *Synechocystis* 6803 [7] to evaluate the degree of GG^4m^CC methylation on plasmid pSYSA (Fig. 1). Bisulfite sequencing permits the direct and highly sensitive detection of 5-methylcytosines, but it can also be used to map 4-methylcytosines; although ^m4^C is partially resistant to bisulfite-mediated deamination and therefore the assay is less sensitive [31]. There are 246 GGCC motifs on plasmid pSYSA per DNA strand, one every 418 nt. The measured GG^4m^CC methylation was 42% on average (Fig. 1c). This value matches the reported average sensitivity for the detection of this DNA modification by bisulfite sequencing [31]. The specificity of the detected GG^4m^CC methylation was verified by analyzing a *sll0729* deletion mutant lacking M.Ssp6803II activity in parallel, where we found zero methylation at these sites (not shown). The GGCC sites are relatively randomly distributed on pSYSA with one important exception, a region entirely free of this motif ranging from position 3537 to position 14,544 (Fig. 1b). The GGCC-free region starts in the gene *ssl7007* encoding the toxin component of a toxin-antitoxin system [32] and finishes within *slr7015* (*cas4*) (Fig. 1d). Hence, this stretch encompasses all the genes belonging to the iCas and pCas functional modules according to the classification suggested by Roger Garrett [33] and separating them from the *cas4–1-2* cassette making up the aCas module for adaptation. The GGCC-free sequence also contains *slr7008* encoding an IS4 transposase and *sll7009* encoding a transcriptional repressor [34] that possesses a WYL domain [35].

### High methylation of CRISPR1 repeats and hypomethylation of *cas* genes at CGATCG sites

**Fig. 2:**
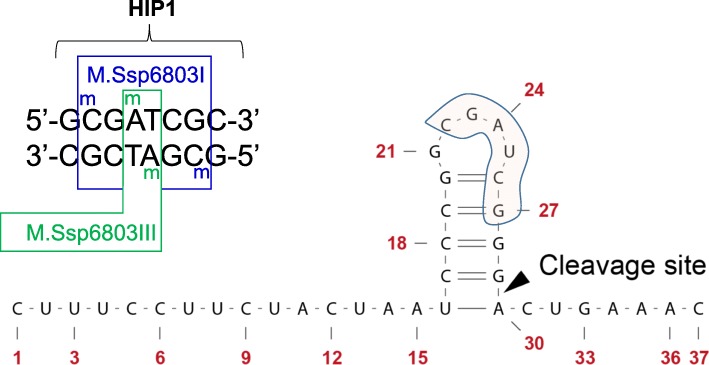
Four DNA methylation events can occur within the 5′- CGATCG − 3′ sequence that is at the core of the octameric HIP1 element. These methylations are carried out by the enzymes M.Ssp6803I (blue) and M.Ssp6803III (green) [7]. A 5′- CGATCG − 3′ recognition sequence is located within every single repeat of the *Synechocystis* 6803 CRISPR1 system. After transcription, this recognition sequence corresponds to four of the five nucleotides within the crRNA loop and to the two upper nucleotides of the right arm within the repeat stem-loop secondary structure that is essential for recognition by the Cas6–1 endoribonuclease, cleaving at the indicated site [20]

We detected one CGATCG sequence that is recognized by the DNA methyltransferases M.Ssp6803I and M.Ssp6803III (recognizing the internal GATC [7]) within every single of the CRISPR1 repeats of *Synechocystis* 6803. All motif instances are preceded by a G residue, hence matching seven of the eight nucleotides of the HIP element (GCGATCGC) (Fig. 2). After transcription, the CRISPR repeats are recognized by processing maturases, which frequently belong to the Cas6 class of endoribonucleases [16]. This maturation step is obligatory for successful interference and often involves recognition of a repeat-internal stem-loop secondary structure [36]. In case of the *Synechocystis* 6803 CRISPR1 system this step is performed by the Cas6–1 enzyme [14, 20]. Therefore, we judged the location of the repeated CGATCG sequences with regard to the repeat secondary structure after transcription. The repeat-internal CGATCG motifs after transcription form 4 of the 5 unpaired loop nucleotides and two nucleotides of the right arm of the RNA stem (Fig. 2). Hence, this motif is part of the sequence that has been found to be functionally relevant for subsequent recognition by the Cas6–1 maturation endonuclease.

**Fig. 3:**
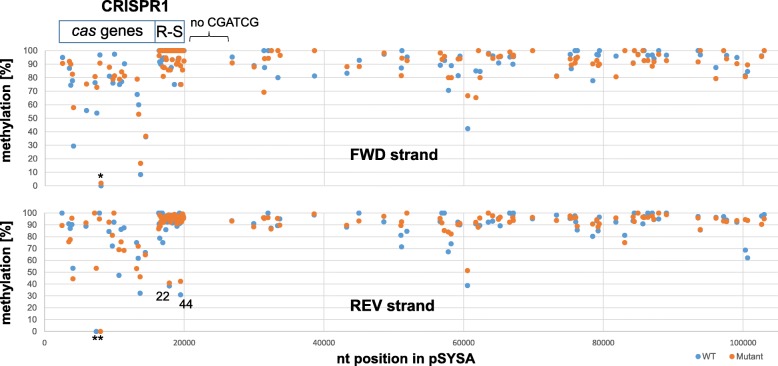
DNA ^5m^CGATCG methylation of the plasmid pSYSA in *Synechocystis* 6803 measured by bisulfite analysis. Percentages of ^5m^CGATCG methylation are plotted for the forward strand (upper panel) and reverse strand (lower panel) of the pSYSA plasmid. Duplicate analyses were performed, using DNA from the wild-type (WT) strain (blue circles) and a ∆*sll0729* mutant (orange circles) that does not impact ^5m^CGATCG methylation. The location of *cas* genes and the repeat-spacer array (R-S) is given on top. The asterisks label two undermethylated motifs within the gene *slr7010*. In the *Synechocystis* 6803 reference strain pSYSA is 103,307 nt long [51] and the CRISPR1 repeat-spacer array extends from positions 16,310 to 19,901 and consists of 50 repeats. The segment from position 19,901 to 26,797 contains no CGATCG sites. Methylation is lower for repeat instances 22 and 44 on the reverse strand

The CRISPR1 system of *Synechocystis* 6803 consists of 50 repeats and 49 spacers in the reference strain [14]. The motif CGATCG contains two methylated bases, the first cytosine methylated by M.Ssp6803I and the adenosine methylated by M.Ssp6803III, together yielding ^5m^CG^6m^ATCG. Because the motif is palindromic, there are four possible methylation events per CGATCG within the DNA double strand. Hence, over a stretch of only 3600 nt, there are 200 possible methylation events for the DNA double strand due to the CRISPR1 repeats alone. Moreover, the CRISPR1 repeat-spacer array contains within its spacer sequences four GGCC and eight GATC sites, which are not part of the CGATCG motif, possibly containing additional methylated bases. This very tight clustering of DNA methyltransferase recognition sites might lead to an undermethylation when the enzyme was locally limiting, but this was not observed. On average, the methylation level of ^5m^CGATCG measured by bisulfite sequencing for the entire pSYSA forward strand was 90.5 and 88.6% for the reverse strand and it was especially high in the repeat-spacer region (Fig. 3). We used two different strains for this analysis, the wild type and a ∆*sll0729* mutant that lacks M.Ssp6803II activity but not affecting ^5m^CGATCG methylation. The measured methylation level of ^5m^CGATCG in the repeat-spacer region reached 97.6 and 96% for the forward strand and 92.5 and 93% for the reverse strand each in the wild type and ∆*sll0729* mutant, respectively (*n* = 50). We conclude that the level of ^5m^CGATCG methylation in this region was close to saturation on the forward strand and on the reverse strand with the exception of repeats 22 and 44 as well (Fig. 3). The plasmid pSYSA exists in multiple copies, likely similar to the chromosome for which ~ 20 copies per cell were reported during exponential growth phase [37]. Therefore, there was a high chance of freshly replicated plasmid copies explaining the average methylation below 100%.

**Fig. 4:**
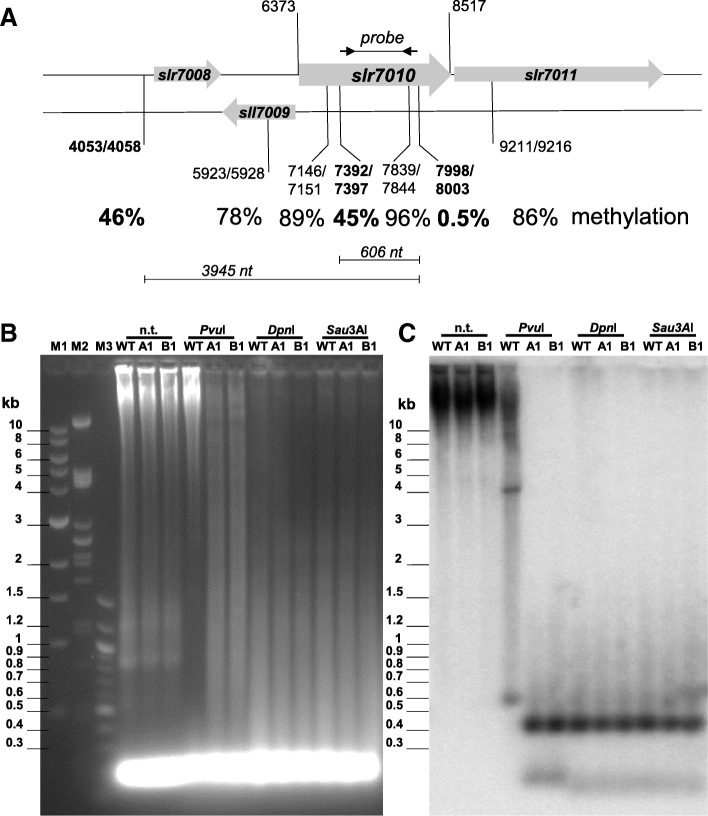
Validation of ^5m^CGATCG methylation status by Southern blot hybridization. **a** Scheme of the probed region from the CRISPR1 system of *Synechocystis* 6803 plasmid pSYSA and percentage of methylated cytosine residues at the indicated positions (first number, position on forward strand; second number, position on the reverse strand) according to bisulfite analysis. Undermethylated sites are bold-faced. The location of primers to generate the 600 bp probe for hybridization to *slr7010* DNA fragments is indicated by arrows. **b** Gel image of the DNA from wild type (WT) and the two *slr0214* mutants (A1 and B1) with no treatment (n.t.) and after restriction by *Pvu*I, *Dpn*I or *Sau*3AI and separation by agarose gel electrophoresis. Three different markers were used as size standards, a 1 kb (M1) and a 100 nt –ladder (M3) and DNA of bacteriophage λ after restriction by *Pst*I (M2). **c** Image of the blot resulting from the gel in panel (**b**) after Southern transfer and hybridization to the probe indicated in panel (**a**). The lengths of the two additional bands in *Pvu*I-digested DNA from the wild type correspond to the predicted lengths indicated in panel (A) for the products of partial digestion between the sites at positions 4053/4058 or 7392/7397 and 7998/8003 due to the different methylation levels

However, we noticed striking imbalances of ^5m^CGATCG methylation levels with regard to the CRISPR1 *cas* genes. The region encompassing all *cas* genes (from nt position 5000 to 16,100) showed hypomethylation of this motif with only 63.4 and 66.3% methylation for the forward and reverse strand). Methylation was nearly absent at the motif positions 7392/7397 and 7998/8003 (Figs. 3 and 4). These positions reside within the *slr7010* gene encoding the Cas3’ protein (Fig. 4).

### Verification of CGATCG hypomethylation by southern hybridization

The ^m5^C hypomethylation at the CGATCG sites detected for the CRISPR1 *cas* genes by bisulfite sequencing was independently tested by Southern hybridization. Total DNA was isolated from the wild type, the *slr0214* deletion mutant (Δ*slr0214*-A1) and the *slr0214* insertion mutant (Δ*slr0214*-B1). For restriction analysis, *Pvu*I was chosen that cuts CGATCG but is sensitive to the methylated ^m5^CGATCG site. Hence, *Pvu*I can only cleave when the cytosine in CGATCG is unmethylated, but it is not affected by methylation of the internal adenosine. For analysis, we chose the *slr7010* gene encoding the Cas3’ protein (Fig. 1). According to bisulfite analysis, CGATCG at positions 7392/7397 and 7998/8003 were nearly not ^m5^C methylated, while the interspersed CGATCG at position 7839/7844 was methylated. Treatment by *Pvu*I yielded two fragments, 447 bp and 159 bp in size with DNA from the two *slr0214* mutants (Fig. 4), confirming the unmethylated status of all relevant sites. With DNA from the wild-type strain, these products were not obtained but a signal at ~ 600 bp, which corresponds to the 606 bp fragment consisting of both parts. Hence, this result validates the bisulfite analysis with mainly cytosine-unmethylated CGATCG sites at positions 7392/7397 and 7998/8003, while the interspersed CGATCG at position 7839/7844 was methylated. In addition, a band at ~ 3.9 kb was detected, which corresponds to another restriction fragment from position 4053/4058 to 7998/8003 (Fig. 4a). According to bisulfite sequencing, the cytosines at 4053/4058 were methylated at 46 ± 12.7% (*n* = 4). For control, aliquots of the same DNA samples were digested by *Dpn*I and *Sau*3AI. *Dpn*I cleaves only when its recognition site is Dam-methylated, G^m6^ATC, whereas *Sau*3AI is insensitive to any of these methylations. The resulting restriction fragments were of identical lengths in all three samples, pointing to full G^m6^ATC methylation. The smaller fragment was somewhat shorter than the 159 bp fragment generated by *Pvu*I, which is due to an internal GATC site, leading to a 30 bp shorter fragment.

### Missing ^m5^CGATCG methylation affects conjugation rates but not CRISPR1-mediated DNA interference

Within this study, we identified specific imbalances in the distribution and methylation level of GGCC and CGATCG sites within the CRISPR1 system of *Synechocystis* 6803. The tightly clustered appearance of many methylated bases within the repeat-spacer array could affect DNA interference. Therefore, we performed assays for conjugation and interference efficiency in wild type and the two different *slr0214* mutant lines that lack the methylation activity of M.Ssp6803I encoded by this gene.

**Fig. 5:**
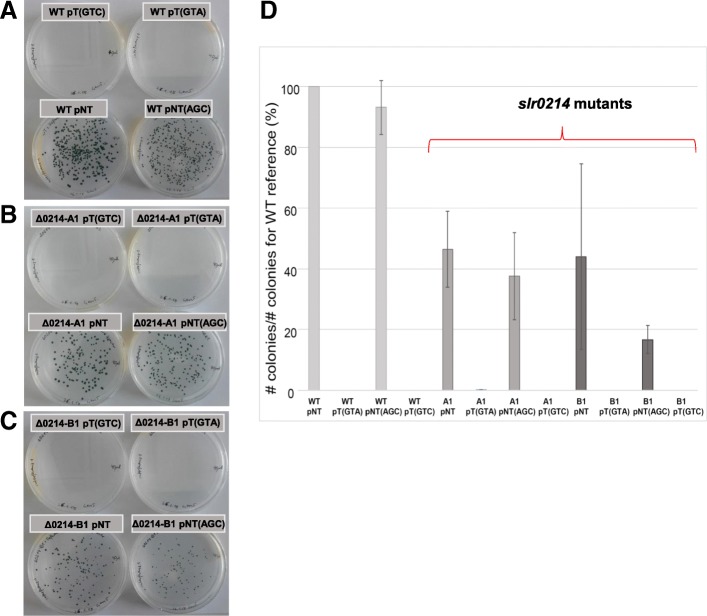
Assays for conjugation and interference efficiency in wild type (WT) and *slr0214* mutant cell lines. The conjugation efficiency was tested for plasmids carrying a protospacer to spacer 1 and a functional protospacer adjacent motif (PAM) of the CRISPR1 system and therefore were potential targets (labelled pT) or that lacked the protospacer or maintained a non-PAM fused to the protospacer and served as non-target controls (labelled pNT). The protospacer in the pT plasmids was linked to a functional GTC or GTA PAM that facilitates recognition of the invader DNA or to an AGC non-PAM that does not license interference in *Synechocystis* 6803 [24]. **a** Plates showing interference and growth of colonies following conjugation into wild type cells. **b** Assay in the Δ*slr0214*-A1 deletion mutant. **c** Assay in the Δ*slr0214*-B1 insertion mutant. **d** Conjugation efficiencies (numbers of colonies normalized to the wild type with the pNT plasmid control). The bars represent the mean ± SE of three biological replicates each

Due to the lack of identified bacteriophages infecting *Synechocystis* 6803, we used a conjugation-based assay. To trigger interference, we inserted a protospacer into the conjugative vector matching spacer 1 of the CRISPR1 system. In addition, we added different PAM sequences that should facilitate the differentiation between “self” and “non-self” targets [24]. The CRISPR1-mediated DNA interference was as efficiently in the two different Δ*slr0214* mutants as in the wild type control and no difference was observed regarding the different PAMs (Fig. 5a-c). In all combinations, the GTC and GTA PAMs facilitated DNA interference, while the AGC non-PAM did not and served as a control. We conclude that the absence of CRISPR1 ^m5^C methylation had no effect on the efficiency of DNA interference. However, a difference was noticed in the conjugation efficiencies, which were in all instances below 50% compared to the wild type (Fig. 5d).

## Discussion

In this work, we focused on the differential methylation on the plasmid pSYSA, but DNA methyltransferases can target any of their recognition sequences on the chromosome as well as on the other six plasmids. *Synechocystis* 6803 encodes five DNA methyltransferases. Two of these are encoded on plasmid pSYSX (M.Ssp6803IV and M.Ssp6803V) and three on the chromosome [7]. Among these DNA methyltransferases, M.Ssp6803I executes ^m5^CGATCG methylation, M.Ssp6803II performs GG^4m^CC and M.Ssp6803III the dam-like G^m6^ATC methylation [7]. Missing cytosine N4-methylation of GGCC motifs in the ∆*sll0729* mutant that lacks M.Ssp6803II activity resulted in strong phenotypical alterations, which were associated with the regulation of transcription, DNA replication and DNA repair [38]. For the methylation of the HIP1-related motif via M.Ssp6803I, a role in DNA repair processes was previously suggested [5]. With regard to transformation, it was reported that the methylation of plasmid DNA in *E. coli* expressing M.Ssp6803I encoded by the *Synechocystis* 6803 gene *slr0214* prior to transformation led to an 11- to 161-fold-higher efficiency in the subsequent integrative transformation of *Synechocystis* 6803 [39]. In contrast, expression of M.Ssp6803II from gene *sll0729* methylating GGCC [7] had no measurable impact on transformation efficiencies [39]. Both of these DNA methyltransferases do not belong to a restriction-modification system. Therefore, the molecular basis for these observations has remained elusive.

During the study of methylation frequencies of single CGATCG sites, we made the remarkable observation that only a few sites were almost unmethylated, while the vast majority of sites was methylated almost 100%. One virtually methyl-free CGATCG site is situated on the plasmid pSYSA within the CRISPR-Cas systems encoding genes, which raised our attention and initiated the detailed functional study reported here. CRISPR-Cas systems involve DNA recombination at their very heart. Degradation products of the RecBCD repair complex were found to serve as templates for spacer acquisition as new spacers [40], especially in the naïve spacer acquisition in *E. coli* [41], with the RecBCD helicase function as the most important activity [42]. However, also vice versa, *cas1* gene deletions were found to affect chromosome segregation and to lead to increased sensitivity towards DNA damage [43]. Another, recently discovered, bacterial phage resistance system called bacteriophage exclusion (BREX) [44] distinguishes self from non-self DNA by methylation of a specific DNA site [45]. It should be noticed that the extended GCGATCGG motif present in CRISPR1 repeats constitutes a 1-nt deviation from the cognate HIP1 sequence but left the recognition sequence for the DNA methyltransferase M.Ssp6803III intact. For all these reasons, the connection between DNA methylation and the CRISPR-Cas apparatus is worth investigation.

We noticed a striking absence of GGCC sites among the iCas and pCas modules of the CRISPR1 system. The *cas3’, cas10d, cas7, cas5* and *cas6–1* genes, together with the genes *sll7009* and *slr7008* for a WYL-domain regulator and a transposase, respectively, lack any GGCC site, which may indicate that they originate from another organism, possibly be transferred via horizontal gene transfer. Because this system belongs to the subtype I-D, this observation fits to the previously proposed hybrid character of this subtype, in which signature genes for a type I-C system were combined with a distinct type III gene arrangement [46].

Moreover, we show that the CRISPR1 system of *Synechocystis* 6803 contains M.Ssp6803III recognition sequences within its repeat sequences and that they are highly methylated while the associated *cas* genes showed hypomethylation at certain but not all ^m5^CGATCG sites. We verified the lowered methylation within the *slr7010* gene encoding Cas3’ for the positions 7392/7397 and 7998/8003 by Southern hybridization while the motif at position 7839/7844 was methylated. The reduced or lacking DNA methylation detected in this work might be related to the binding of one or several regulatory factors. We noticed that the undermethylated sites within *slr7010* are located next to *sll7009* that upon deletion caused an enhanced CRISPR1 expression leading to the classification of Sll7009 as a repressor [34].

We then asked whether the lacking methylation due to mutation of *slr0214* would have an impact on the highly efficient DNA interference associated with this system but this was not the case. Moreover, the high transcription of the CRISPR1 repeat-spacer array and its dense and quantitatively high methylation obviously did not interfere with each other. This matches reports for HIP1 to not play a direct role in the regulation of gene expression [4].

However, we detected an approximately 50% reduced conjugation efficiency in the Δ*slr0214* mutant compared to the wild type. But, DNA methylation is not restricted to plasmid pSYSA containing the CRISPR system, hence, there is no reason to assume that the lack of M.Ssp6803I–mediated methylation on pSYSA was the only causative for the observed reduced conjugation efficiency. The detected correlation between the genome-wide lacking ^m5^CGATCG DNA methylation and the reduced conjugation efficiency cannot be related to DNA integration and recombination because we used a conjugative plasmid that replicates in the cell autonomously and does not require DNA recombination of integration. Moreover, it cannot be related to DNA restriction because M.Ssp6803I is not part of an RM system [7]. Generally, DNA methylation in bacteria can contribute to the transcriptional regulation of genes involved in diverse processes, ranging from biofilm formation, bacteriophage replication, transposition, the timing of chromosome replication and mismatch-repair to conjugation [47]. Therefore it is highly intriguing that instances were described in which DNA methylation affect conjugative transfer, e.g., of a plasmid in *Salmonella enterica* [48]. In that system, transcription of *traJ* is increased while transcription of *finP* that antagonizes *traJ* expression, is reduced in a *dam* mutant. The ratio between the methylation and transcription of these two genes accounts for the level of *tra* operon expression and the efficiency of conjugation of that plasmid [49, 50]. Lacking DNA methylation leads to measurable changes in gene expression in *Synechocystis* 6803 as well [38]. Therefore, it is not unlikely that a yet unknown regulatory process that is affected via changed gene expression in mutant Δ*slr0214* possibly is also affecting plasmid uptake or replication resulting in lowered conjugative efficiency.

## Conclusions

The majority of bacteria and archaea use CRISPR-Cas systems for antiviral defense. Multiple relationships between DNA methylation, DNA recombination, repair and CRISPR-Cas systems were previously reported. Here, we addressed the possibility of directs links between DNA methylation and the CRISPR-Cas apparatus using the cyanobacterium *Synechocystis* 6803 as a model. Major DNA methylation sites in this organism are the GG^4m^CC motif recognized by M.Ssp6803II and the HIP1-related motif ^5m^CG^6m^ATCG recognized by M.Ssp6803I and M.Ssp6803III, respectively. We report a remarkable discrepancy in the distribution of GGCC sites along the CRISPR1 *cas* genes, supporting the hybrid character of subtype I-D CRISPR-Cas systems. The here identified tight and very high ^5m^C methylation of the CRISPR1 repeat sequences point at some functional relevance. Indeed, while cells lacking the ^5m^C methylation activity were unaffected in the CRISPR1-mediated interference response, the efficiency of conjugation was reduced to ~ 50%. Because we used a conjugative plasmid to challenge the CRISPR1 system, the observed difference cannot be related to DNA integration and recombination as might have been involved when integrative transformation of a suicide vector was used as a read-out [39]. Instead, the results point to an unknown role of ^m5^CGATCG DNA-methylation marks in conjugation and DNA transformation.

## Data Availability

Previously generated bisulfite raw data re-analyzed during the current study are available at https://www.ncbi.nlm.nih.gov/biosample/8378604 (BioProject ID: PRJNA430784, BioSample: SAMN08378604, Run: SRX3574087). All other data generated or analyzed during this study are included in this published article.

## Abbreviations

aCas: adaptation Cas
Cas: CRISPR-associated proteins
CRISPR: Clustered regularly interspaced short palindromic repeats
crRNAs: CRISPR RNAs
HIP1: Highly Iterated Palindrome 1
iCas: interference Cas
PAM: Protospacer adjacent motif
pCas: processing Cas
